# Cholesterol Conjugates of Small Interfering RNA: Linkers and Patterns of Modification

**DOI:** 10.3390/molecules29040786

**Published:** 2024-02-08

**Authors:** Ivan V. Chernikov, Ul’yana A. Ponomareva, Mariya I. Meschaninova, Irina K. Bachkova, Valentin V. Vlassov, Marina A. Zenkova, Elena L. Chernolovskaya

**Affiliations:** 1 Institute of Chemical Biology and Fundamental Medicine, Siberian Branch of the Russian Academy of Sciences, Academic Lavrentiev Avenue 8, 630090 Novosibirsk, Russia; chernikovivanv@gmail.com (I.V.C.); uljana@ngs.ru (U.A.P.); mesch@niboch.nsc.ru (M.I.M.); i.bachkova@g.nsu.ru (I.K.B.); marzen@niboch.nsc.ru (M.A.Z.); 2 Faculty of Natural Sciences, Novosibirsk State University, Pirogova Str. 1, 630090 Novosibirsk, Russia

**Keywords:** siRNA, chemical modifications, cholesterol conjugate, nuclease resistance, duration of silencing, *MDR1* gene

## Abstract

Cholesterol siRNA conjugates attract attention because they allow the delivery of siRNA into cells without the use of transfection agents. In this study, we compared the efficacy and duration of silencing induced by cholesterol conjugates of selectively and totally modified siRNAs and their heteroduplexes of the same sequence and explored the impact of linker length between the 3′ end of the sense strand of siRNA and cholesterol on the silencing activity of “light” and “heavy” modified siRNAs. All 3′-cholesterol conjugates were equally active under transfection, but the conjugate with a C3 linker was less active than those with longer linkers (C8 and C15) in a carrier-free mode. At the same time, they were significantly inferior in activity to the 5′-cholesterol conjugate. Shortening the sense strand carrying cholesterol by two nucleotides from the 3′-end did not have a significant effect on the activity of the conjugate. Replacing the antisense strand or both strands with fully modified ones had a significant effect on silencing as well as improving the duration in transfection-mediated and carrier-free modes. A significant 78% suppression of *MDR1* gene expression in KB-8-5 xenograft tumors developed in mice promises an advantage from the use of fully modified siRNA cholesterol conjugates in combination chemotherapy.

## 1. Introduction

RNA interference (RNAi) is an evolutionarily conserved mechanism present in the cells of all eukaryotes that implements sequence-specific suppression of gene expression at the mRNA level [1]. Small interfering RNAs (siRNAs) are the inducers of RNAi that can be obtained synthetically and used for research and clinical purposes [2,3]. Efficient delivery of siRNA into cells and stability against nucleases are the key factors for the development of RNAi-based therapeutic strategies. Currently, five siRNA-based drugs for the treatment of liver-related diseases have successfully passed clinical trials and are approved for use in clinical practice [4,5,6,7]. The delivery of siRNA to other organs still remains a challenge. Conjugation of siRNA with carrier molecules represents the most promising approach among siRNA delivery systems, since siRNA conjugates exhibit minimal toxicity in vivo and favorable biodistribution. Folate [8], carbohydrates [9,10], cholesterol [11], aptamers [12], peptides [13,14], antibodies [15], lipids, and aliphatic chains of varying lengths [16] have been used for the attachment to siRNA [7,17]. The conjugation of siRNA with cholesterol is one of the most favorable and provides enhanced accumulation of siRNA in various extrahepatic organs such as tumors, placenta, muscles, heart, and others [18,19,20,21,22,23,24].

siRNA is more or less protected from nuclease degradation when delivered as part of various complexes with cationic lipids, polymers, or particles, but siRNA within a conjugate with a transport molecule remains available for ribonucleases’ cleavage [25]. Therefore, this approach could be used only in combination with chemical modifications that stabilize siRNA in the presence of ribonucleases. However, chemical modifications of the ribose-phosphate backbone can affect the RNA interference process, necessitating a balance between the number of modifications and the efficiency of RNAi. The tolerance of the RNAi system to modification of different positions in the antisense and sense strands is being actively studied [26,27,28]. Previously, our laboratory developed a highly efficient, selectively modified anti-*MDR1* siRNA with 2′OMe modifications in nuclease-sensitive sites. It was shown that selectively modified siRNA and its cholesterol conjugates are significantly more stable in serum and induce long-lasting gene silencing compared to their unmodified counterparts [29]. This pattern belongs to the so-called “light” modification patterns. Later on in the study [30], a pattern of chemical modifications containing 2′F, 2′OMe, and PS modifications of all nucleotides and providing prolonged efficacy of GalNAc-siRNA conjugates in vivo was developed. This pattern represents itself as a pattern of “heavy” modifications. However, the influence of the siRNA chemical modification patterns in the composition of cholesterol conjugates on its bioperformance and duration of silencing effect has not been systematically studied.

The nature, length, and location of the linker attachment point can also have a significant impact on the accumulation and silencing activity of the siRNA conjugate. Previously, we have shown that the linker length between the 5′ end of the sense strand of siRNA and cholesterol residue is an important factor affecting the accumulation and silencing activity of siRNA [31]. An increase in linker length from three to six carbon atoms resulted in improved accumulation and silencing activity of siRNA; a further increase in linker length to twelve carbon atoms increased accumulation but decreased silencing activity. The influence of linker length on the silencing activity of siRNA conjugated to cholesterol via 3′-end has not been investigated.

Therefore, in this study, we compared the efficacy and duration of silencing induced by cholesterol conjugates of selectively and totally modified siRNAs and their heteroduplexes of the same sequence, and explored the impact of linker length between the 3′ end of the sense strand of siRNA and cholesterol on the silencing activity of “light” and “heavy” modified siRNAs.

## 2. Results

### 2.1. Silencing Activity of Cholesterol-Modified siRNA under Transfection

**Figure 1:**
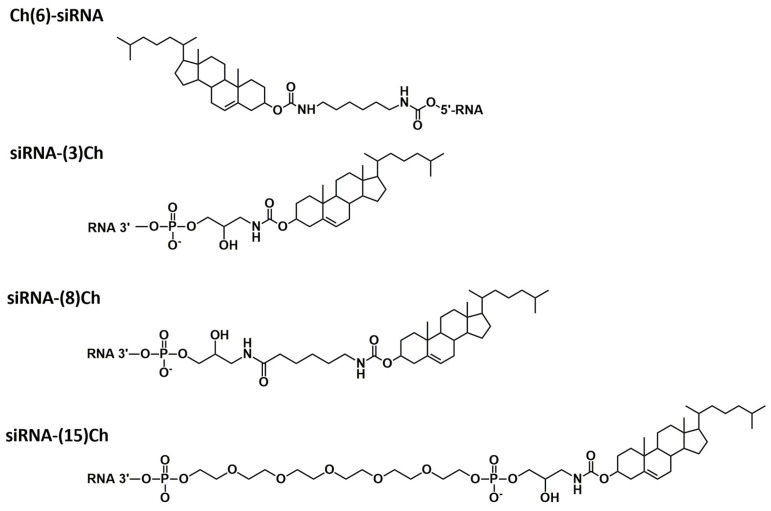
Schematic structure of the conjugates.

A series of siRNAs and their cholesterol conjugates (Ch-RNA) aimed at suppressing the expression of the *MDR1* gene were designed and studied on a model KB-8-5-MDR1-GFP cell line expressing MDR1-GFP chimeric bicistronic mRNA [31,32]. We selected this transport ligand since the attachment of cholesterol allows its conjugate with siRNA to effectively penetrate into the cell and participate in RNA interference more effectively than other lipophilic ligands [31]; intramuscular or subcutaneous injection of cholesterol conjugate ensures retention of the conjugate at the site of local administration and its slow spread [18]. Selectively modified siRNAs (light pattern) contained 2′OMe modifications of CpA, UpA, and UpG nuclease-sensitive sites, according to the algorithm we described earlier [29], while fully modified siRNAs (heavy pattern, designated as FM) contained 2′F and 2′Ome modifications distributed along siRNA by analogy with the pattern described in [30], except that PS modifications were not applied (Table 1). Two types of duplexes were used in the work: classic 21/21 nt with two overhanging nucleotides at the 3′-ends of the strands, and truncated 19/21 nt duplexes with a blunt end at the 3′-end of the sense strand (designated as −2G), since such structures are described in numerous publications devoted to conjugates [20,23,33,34,35,36]. The asymmetrical structure of the duplex can affect the asymmetric assembly of the RNA-induced silencing complex (RISC), as well as the distance from the transport molecule to this complex and, accordingly, the choice of linker. Cholesterol, as a transport molecule, was attached to the 5′- or 3′-ends of the sense strand (Figure 1). The 5′-Cholesterol conjugated siRNA contained a hexamethylenediamine linker selected previously as optimal for both accumulation and silencing (Ch(6)5′-siMDR1) [18,32]. The 3′-cholesterol conjugated siRNA contained linkers of different lengths: the shortest linker was based on serinol (siMDR1-(3)Ch), then the serinol linker was extended by aminohexanoic acid (siMDR1-(8)Ch), and the longest linker was based on hexaethylene glycol extended by serinol (siMDR1-(15)Ch) (Figure 1). Lipofectamine-mediated delivery of siRNA conjugated into cells was used to evaluate the effect of the introduced modifications on the silencing activity of the conjugates and to exclude the influence of the modifications on the efficiency of intracellular accumulation, which manifests itself when delivered in a carrier-free mode.

**Figure 2:**
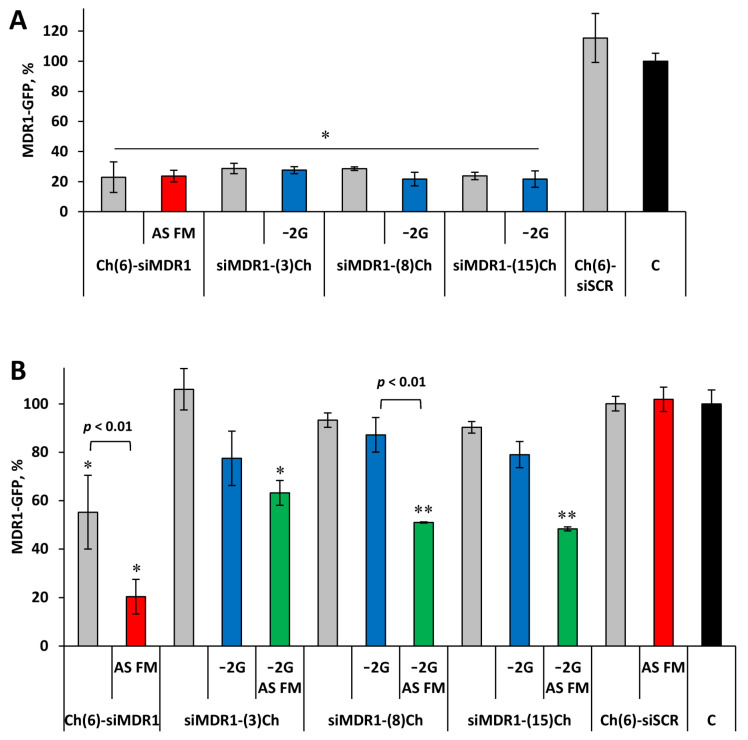
Silencing of *MDR1-GFP* gene expression in KB-8-5-MDR1-GFP cells by siRNA conjugates. Flow cytometry data obtained at 72 h following 100 nM conjugate transfection by Lipofectamine 2000 (**A**) or 5 μM conjugate delivery in a carrier-free mode (**B**). C—control, untreated cells. Gray bars—21/21 selectively modified siRNA, blue bars—19/21 selectively modified siRNA, green bars—19/21 siRNA with fully modified antisense strand, red bars—21/21 siRNA with fully modified antisense strand. Mean values (±SD) and statistical significance of differences from control (*—*p* < 0.05, **—*p* < 0.01), calculated using Student’s *t*-test from the results of three independent experiments, are shown in the figure.

The results show that upon lipofection, all siRNAs (100 nM) suppress the expression of the target gene with similar efficiency: MDR1-GFP expression was reduced to 22–29% of the level in control or siScr-treated cells (Figure 2). No significant effect of the linker length at the 3′ end of the siRNA sense strand, as well as no significant differences between the effectiveness of the 3′- and 5′-conjugates, were found. The presence of overhangs also did not affect the silencing activity of siRNA: target mRNA levels were 22–28% and 24–29% for the 21/21 and 19/21 siRNA, respectively (Figure 2A). Additional 2′OMe/2′F modifications into the antisense strand of siRNA (AS_FM) did not affect the interfering properties of siRNA—23 and 24% for Ch(6)-siMDR1 and Ch(6)-siMDR1_FM, respectively. Thus, it can be assumed that the studied modifications do not prevent RNA interference under transfection conditions.

### 2.2. Silencing Activity of Cholesterol-Modified siRNA in Carrier-Free Mode

The influence of the linker length and attachment point on the silencing activity of Ch-RNA in a carrier-free mode was analyzed under the same conditions, but the concentration of the conjugates was 5 μM, since it was previously shown that 5′-cholesterol conjugates effectively suppress the expression of MDR1-GFP at this concentration [31]. It was found that, unlike 5′-conjugates (45% silencing), all selectively modified 3′-cholesterol conjugates of 21/21 siRNA did not exert a silencing effect on the target gene expression (Figure 2B, gray bars). At the same time, 3′-conjugates lacking two protruding nucleotides at the 3′-end of the sense strand (−2G) tended to induce minor silencing, reducing target gene expression by 13–23%, but these differences were not statistically significant (Figure 2B, blue bars).

Replacement of the antisense strand in the studied duplexes with fully 2′OMe/2′F modified ones (AS FM) had a positive effect on the silencing activity of the conjugates, additionally increasing the silencing effect by 14–36%, resulting in 47–52% silencing (Figure 2B, green bars). An even more pronounced dependence of silencing activity on the location of cholesterol residue (3′ or 5′) was observed for these conjugates: 80% for 5′- and not more than 52% for 3′-conjugates (*p* < 0.05). In addition, a moderate difference in the activity of conjugates containing linkers of different lengths was revealed under carrier-free conditions. The conjugate with a relatively short serinol linker of approximately three carbon atoms between siRNA and cholesterol silenced target gene expression by 37%. By extending the serinol-based linker with aminohexanoic acid to approximately eight carbon atoms, the silencing activity of the conjugate increased to 49% (siMDR1-(8)Ch−2G_AS_FM vs. Control, *p* < 0.01, Figure 2B). Further extension of the linker to approximately 15 methylene units (hexaethylene glycol linker) did not change the silencing (52%, siMDR1-(15)Ch−2G_AS_FM vs. Control, *p* < 0.01, Figure 2B). It is worth mentioning that changing the siRNA-Ch design—shortening the sense strand (−2G) and increasing linker length, as well as using heavy modifications of the antisense strand—made it possible to achieve silencing under the action of 3′-cholesterol conjugates, which were previously inactive.

The activity of the 5′-cholesterol conjugate when the antisense strand was replaced with a fully modified one increased even more significantly: the silencing effect amounted to 45% and 80% for Ch(6)-siMDR1 and Ch(6)-siMDR1_AS_FM, respectively (*p* < 0.01 between them). Thus, Ch(6)-siMDR1_AS_FM had the highest silencing activity among the studied cholesterol-containing siRNAs. These data allow us to conclude that significant differences in silencing activity in a carrier-free mode are associated with differing efficiency of productive penetration of conjugates with different structures into cells, since the activities of the conjugates under transfection with Lipofectamine were comparable.

### 2.3. Silencing Activity of Selectively and Fully Modified siRNA

2′OMe/2′F modifications have a significant effect on the activity of cholesterol conjugates when delivered without a carrier; therefore, we compared dose-dependent inhibition of the target mRNA by selectively modified siRNAs, heteroduplexes with a fully modified antisense strand, and duplexes with both strands fully modified to evaluate the concentration at which MDR1-GFP gene expression decreases by 50% (IC_50_) under transfection with Lipofectamine. We showed that total 2′OMe/2′F modification of the antisense strand and both strands reduced the IC_50_ of siRNA by 4- and 9-fold, respectively (Table 2, *p* < 0.01). The attachment of cholesterol to siMDR1 increased the IC_50_ by 3.5 times (Table 2, *p* < 0.05), probably due to the interaction of cholesterol with RNAi proteins. However, similar relationships were found for the 5′-cholesterol conjugates: the total 2′OMe/2′F modification of the antisense strand and both strands of Ch-siMDR1 reduced IC_50_ by 5- (*p* < 0.05) and 50 (*p* < 0.01)-fold, respectively (Table 2). Thus, the introduction of 2′OMe/2′F into both siRNA strands compensated for the negative effect of cholesterol attachment. It should be noted that although the 2′OMe/2′F modifications did not affect the silencing activity of Ch-RNA at 100 nM concentration (Figure 2A), the IC_50_ values presented in Table 2 differ significantly, reflecting a pronounced difference in the activity of the conjugates at low concentrations.

**Figure 3:**
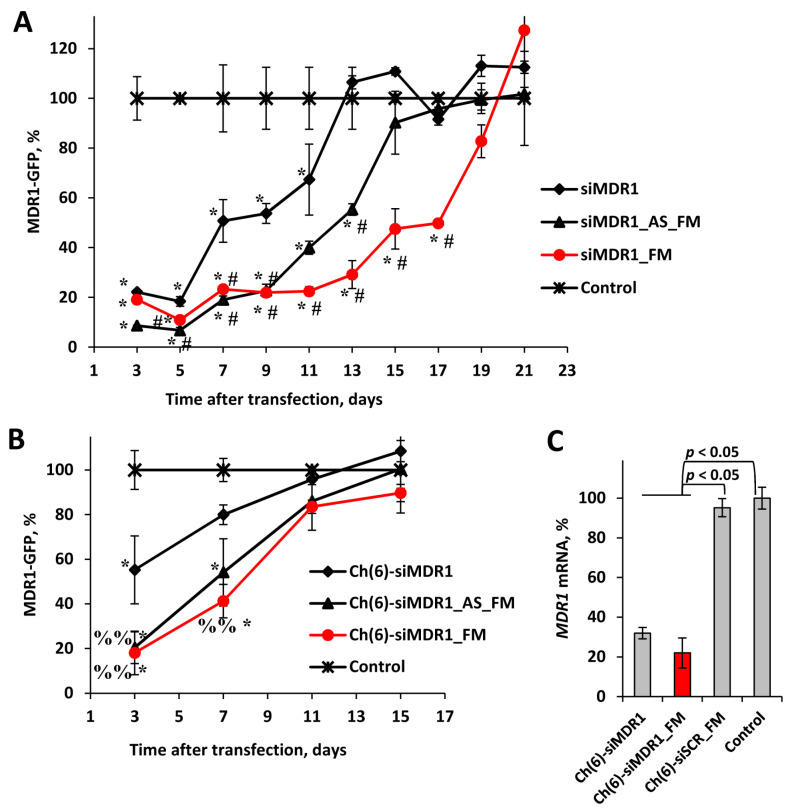
Silencing activity of anti-*MDR1* siRNAs with different modification patterns and their 5′-cholesterol conjugates. (**A**,**B**) The kinetics of the silencing of *MDR1-GFP* gene expression in KB-8-5-MDR1-GFP cells by siRNAs and their conjugates. Flow cytometry data obtained at 3–21 days following 100 nM conjugate transfection by Lipofectamine 2000 (**A**) or 5 μM conjugate delivery in a carrier-free mode (**B**). Mean values (±SD) and statistical significance of differences from control (*—*p* < 0.05), from siMDR1 (#—*p* < 0.05), and from Ch(6)-siMDR1 (%%—*p* < 0.01), calculated using Student’s *t*-test from the results of three independent experiments, are shown in the figure. (**C**) Silencing of *MDR1* mRNA expression by 7.5 μg/g of cholesterol-modified siMDR1 in KB-8-5 xenograft tumor in SCID mice 4 days after IV injection (*n* = 3–5). Statistical significance of differences in qRT-PCR data were obtained with the Mann–Whitney U test.

Chemical modifications aimed at protecting siRNA from degradation by nucleases are intended to increase both the effectiveness and duration of its action; therefore, we studied the kinetics of siRNA silencing activity when delivering selectively modified siRNA (siMDR1), siRNA with a fully modified antisense strand (siMDR1_AS_FM), and fully modified siRNA (siMDR1_FM) using Lipofectamine (Figure 3A).

It was shown that on days 3–5 after transfection that the silencing effect of the siRNAs under study differed slightly and amounted to 76–91% suppression; siMDR1_AS_FM showed the highest activity. In the next 7–9 days, the effectiveness of the inhibitory effect of siMDR1 and its inhibition was no more than 50%, while the activities of siMDR1_AS_FM and siMDR1_FM did not change so noticeably, and their silencing was about 80%. A difference in the efficiency of action between all siRNAs became more pronounced with further incubation (11–19 days): the silencing effect increased in the series siMDR1 < siMDR1_AS_FM < siMDR1_FM. No statistically significant differences from the control cells were found for siMDR1 starting on the 13th day, for siMDR1_AS_FM starting on the 15th day, and for siMDR1_FM starting on the 19th day (Figure 3A). This decrease in the efficiency of the inhibitory effect of all studied siRNAs is associated both with a decrease in the concentration of siRNA during cell division and with the action of ribonucleases that cleave siRNA. The longer silencing activity of siMDR1_FM, compared to other siRNAs, is provided by full modification of all 2′-positions of ribose in the duplex.

The duration of silencing activity of cholesterol-modified siRNA without a transfection agent was studied 3–15 days after addition to cells. It was shown that three days after adding the conjugates to the cells, Ch(6)-siMDR1_AS_FM and Ch(6)-siMDR1_FM with equal efficiency reduced the level of expression of the target gene by 80–82%, while Ch(6)-siMDR1 suppressed target gene expression by only 45% (Figure 3B), which corresponds with the data presented in Figure 2B. A statistically significant difference from the control cells was not found after seven days for Ch(6)-siMDR1 and 11 days for Ch(6)5′-siMDR1_AS_FM and Ch(6)-siMDR1_FM (Figure 3B). These data correlate with data reported by Foster et al. [30], where it was shown that fully modified siRNAs have durable silencing activity. The data obtained confirm the advantage of using fully modified siRNAs in cholesterol conjugates and highlight the role of the high stability of the antisense strand in achieving long-lasting effects when delivered without a carrier.

The effect of 2′OMe/2′F modifications on the silencing activity of cholesterol-modified siRNA was studied on SCID mice with xenograft KB-8-5 tumors. The results showed that Ch(6)-siMDR1 and Ch(6)-siMDR1_FM both demonstrated significant silencing activity against the *MDR1* gene in the tumor four days after intravenous injection (Figure 3C). It has been shown that Ch(6)-siMDR1 reduces *MDR1* mRNA to 32% and Ch(6)-siMDR1_FM to 22% of the level in the tumors of control mice or mice injected with Ch(6)-siSCR_FM, although the differences in activity between Ch(6)-siMDR1 and Ch(6)-siMDR1_FM were not statistically significant at the 4-day time point. These findings confirm the potential utility of fully modified cholesterol-siRNA in targeting *MDR1* gene expression for therapeutic purposes.

## 3. Discussion

Cholesterol siRNA conjugates attract the attention of researchers because they allow the delivery of siRNA into cells without the use of transfection agents. Cholesterol in the composition of such a conjugate performs several functions: it increases the hydrophobicity of the conjugate [33,37], allows the avoidance of rapid removal from the bloodstream through filtration by the kidneys, and provides the opportunity to use the natural mechanisms of cholesterol transport to enter the cell [38]. To achieve this goal, cholesterol is attached to the 3′- or 5′-end of the sense strand, since this type of modification is well tolerated by the RNA interference mechanism. The choice of attachment method is usually determined by the convenience of synthesis; therefore, attachment to the 3′-end via an amino linker on a functionalized CPG is more often used, while no attention is paid to comparing the properties of 3′- and 5′-conjugates. We have previously shown that the 3′-cholesterol conjugate with a linker containing three carbon atoms is less active than the 5′-conjugate with a linker of the same length and significantly less active than the 5′-conjugate with optimized 6-carbon linkers due to non-efficient delivery into cells without a carrier. In this work, we tried to restore the activity of the 3′-conjugate by optimizing the linker to its composition and the structure of the duplex, which can also affect the mutual arrangement of cholesterol and siRNA. The obtained data show that all 3′-cholesterol conjugates were equally active under transfection, but the conjugate with a C3 linker was less active than conjugates with longer linkers (C8 and C15) when used in a carrier-free mode (Figure 2). No direct dependence of silencing after carrier-free delivery on the length of the linker was found; this may be due both to the low activity of delivery into cells under the influence of 3′-cholesterol conjugates and to the higher mobility of the linker attached to the 3′-end of siRNA. At the same time, they were significantly inferior in activity to the 5′-cholesterol conjugate. These findings indicate that the site of attachment of the transport ligand is fundamentally important for the process of its internalization into the cell.

Another option for changing the location of the transport ligand relative to the duplex is to modify duplex structure. Asymmetric duplexes containing only one 3′-overhang are often used in research and have been shown to be effective, with the overhang size varying from 2–3 n to 5 n in hsiRNA [35,39]. Such a structure favors the asymmetric assembly of the RISC, ensuring selective inclusion of the antisense strand in its composition, and also reduces the number of single-stranded regions that are more sensitive to cleavage by nucleases.

Shortening the sense strand carrying cholesterol by two nucleotides from the 3′ end, so that the transport ligand was attached to the blunt end of the duplex, did not have a significant effect on the activity of the conjugate, but there was only some tendency for activity to increase when delivered without a carrier (Figure 2B). The presence of two unpaired nucleotides, which may act as an additional cleavable linker between the 3′ end and the cholesterol, could inhibit silencing in a carrier-free mode if these nucleotides are cleaved by nucleases before siRNA enters the cells; however, there is no reliable data to confirm this assumption. It is probably possible to improve the silencing activity of 3′ conjugates by using deoxyribonucleotide or PS modifications in the overhangs, which are more stable to nucleases [35,40].

In this work, unconjugated siMDR1_FM, transfected with Lipofectamine 2000, reduced the level of target gene expression longer than the cholesterol conjugate Ch(6)-siMDR1_FM delivered into cells without a carrier (Figure 2). The difference in the duration of the inhibitory effect of cholesterol derivatives compared to unconjugated siRNAs may be explained by differences in the method of delivery of siRNAs into cells. Probably, transfection might deliver siRNA with more efficiency into the cytoplasm where RNAi occurs compared to carrier-free uptake. The similarity of the kinetics of Ch(6)-siMDR1_AS_FM and Ch(6)-siMDR1_FM may be due to the fact that the sense strand in Ch(6)-siMDR1_AS_FM is modified by cholesterol and has additional protection from nucleases.

A comparison of the silencing activity of partially and fully modified 3′ cholesterol derivatives of 15/20 hsiRNA was carried out in [41]; it was shown that the fully modified cholesterol conjugate demonstrated similar or improved silencing in vitro and in vivo compared to the partially modified conjugate. However, the hsiRNAs compared in this work differed not only in the degree of modification but also in the 2′OMe/2′F patter and numbers of PS modifications, and they were equipped with an uncleavable phosphate at the 5′-end of the antisense strand [41]. It should be noted that the introduction of additional PS modifications at the ends of the duplex as part of a bioconjugate can significantly increase both the silencing activity of siRNA and the duration of its action [42]. It is not possible to exclude the influence of an increase in the number of PS on the activation of thiol-mediated accumulation in cells, which depends on the number of PS and the type of cells [43].

Selective protection of nuclease-sensitive sites with 2′OMe modifications did not reduce the interfering activity of siRNA and significantly increased the nuclease resistance of siRNA in serum [29]. This pattern of chemical modifications was developed based on a rational search for the minimum required number of modifications to ensure resistance to nucleases in the presence of serum and long-term action in vitro, while the location of modifications depended on the siRNA sequence [29]. The fully modified pattern used in this work was developed by Alnylam Pharmaceuticals by sequentially replacing 2′F with 2′OMe, up to 81% of the modifications in the alternating 2′F/2′O-Me pattern (50/50%) [30]. The C3′-*endo* ribose conformation supports an A-helix, which is necessary for efficient RNAi. Both 2′F and 2′OMe modifications stabilize the C3′-*endo* ribose conformation, but in a different way: 2′F slightly over-winds and 2′OMe slightly under-winds double-stranded RNA [41], therefore, their combination makes it possible to successfully protect positions in which RNAi is not tolerant to 2′OMe [20,44].

Previously, our laboratory showed that an increase in the expression level of the *MDR1* gene by 1.5–2-fold leads to the appearance of multidrug resistance syndrome in cells [45]. Therefore, a decrease in the expression level of this gene by 50% is already therapeutically significant after treating cells with the conjugate selectively modified siRNA (Ch(6)-siMDR1). It was shown that this level of suppression of target gene expression is sufficient for the KB-8-5 cells to overcome resistance to 300 nM vinblastine in vitro [31]. It should be noted that the cholesterol conjugate of fully modified siRNA (Ch(6)-siMDR1_FM) studied in this work reduces the MDR1 expression level by almost 40% more effectively compared to Ch(6)-siMDR1 in vitro. Although there is no significant difference in silencing activity between selectively and fully modified cholesterol siRNA conjugates in vivo, a significant 78% suppression of *MDR1* gene expression in a xenograft tumor suggests an advantage from the use of fully modified siRNA cholesterol conjugates in dynamics sufficient for chemotherapy.

## 4. Materials and Methods

### 4.1. Oligonucleotides

The sense (S) and antisense (AS) siRNA strands were targeted to the *MDR1* gene (557–577 n region of the *MDR1* mRNA, GeneBank #M14758); scramble siRNA (siSCRm), which has no significant homology with human, rat, or mouse mRNA, was used as a control (Table 1). Oligoribonucleotides and their analogs were synthesized by the phosphoramidite method on an automatic ASM-800 synthesizer (Biosset, Novosibirsk, Russia). In the synthesis, 2′-O-TBDMS-protected, 2′-F-, 2′-O-Me-ribophosphoramidites, and CPG polymeric carriers with an attached first nucleoside (Glen Research, Sterling, VA, USA) were used. For the synthesis of siRNA conjugates containing a cholesterol residue with a hexamethylene linker at the 5′-end, a solid-phase synthesis method was used based on the activation of the free 5′-hydroxyl group of a protected polymer-bound oligonucleotide with N,N′-disuccimidyl carbonate (Acros Organics, Geel, Belgium), followed by the interaction with cholesteryl-6-aminohexylcarbamate by analogy with [46]. The 3′-Cholesterol siRNA conjugates were obtained using hexaethylene glycol phosphoramidite (Lumiprobe, Moscow, Russia) and/or a cholesterol-modified polymer carrier synthesized by analogy with [47]. The target products were isolated by preparative gel electrophoresis in 15% polyacrylamide gel (PAAG) under denaturing conditions, followed by elution of the products with a 0.3 M NaClO_4_ solution. The isolated products were desalted on a Sep-Pac C18 cartridge (Waters, Milford, MA, USA) and precipitated with a 2% NaClO_4_ solution in acetone. To obtain duplexes, equimolar concentrations of the sense and antisense siRNA strands were incubated in 30 mM HEPES-KOH (pH 7.4), 100 mM potassium acetate, and 2 mM magnesium acetate at 90 °C for 5 min, with a gradual decrease in temperature to 25 °C for 1 h, and the duplexes were stored at −20 °C.

### 4.2. Cell Cultures

A multiple-drug-resistant human cell line, KB-8-5, growing in the presence of 300 nM vinblastine was generously provided by Prof. M. Gottesman (NIH, Bethesda, MD, USA). The KB-8-5-MDR-GFP cell line expressing the fragment of the *MDR1* mRNA and short-lived turboGFP mRNA in a single transcript was obtained by lentiviral transduction as previously described [32]. The KB-8-5 and KB-8-5-MDR-GFP cells were grown in Dulbecco’s modified Eagle’s medium (DMEM) supplemented with 10% fetal bovine serum (FBS), 300 nM vinblastine, 100 U/mL penicillin, 100 μg/mL streptomycin, and 0.25 μg/mL amphotericin at 37 °C in a humidified atmosphere containing 5% CO_2_/95% air.

### 4.3. Silencing Activity Assay Using Flow Cytometry

One day before the experiment, KB-8-5-MDR-GFP cells were plated in 48-well plates at a density of 2.5 × 10^4^ cells/well. After 24 h, the growth medium was replaced with fresh serum-free DMEM (200 µL/well). The siRNAs were added to the cells in 50 µL of Opti-Mem to give the final concentration of 5 µM. Alternatively, the cells were transfected with siRNAs (0.1–100 nM) using Lipofectamine 2000 (Invitrogene, Carlsbad, CA, USA) according to the manufacturer’s protocol (1 µL per well). Four hours after transfection or addition of siRNA in carrier-free mode, the culture medium was replaced with DMEM containing 10% FBS. Three days post-transfection, the cells were trypsinized and 8000 cells from each sample were analyzed using the NovoCyte flow cytometer (ACEA Biosciences, San Diego, CA, USA). Silencing activity data were obtained using mean fluorescence intensity values of cells measured in relative fluorescent units (RFU) and equation MDR1-GFP (%) = (RFU_sample_ (KB-8-5-MDR1-GFP) − RFU(KB-8-5))/(RFU_control_ (KB-8-5-MDR1-GFP) − RFU(KB-8-5)) × 100%; untreated cells were used as a control.

### 4.4. Mice

All animal procedures were carried out in strict accordance with the recommendations for proper use and care of laboratory animals (ECC Directive 86/609/EEC). The protocol was approved by the Committee on the Ethics of Animal Experiments of the Administration of the Siberian Branch of the Russian Academy of Sciences. The experiments were conducted at the Center for Genetic Resources of Laboratory Animals at the Institute of Cytology and Genetics, Siberian Branch, Russian Academy of Sciences (RFMEFI61914X0005 and RFMEFI62114X0010). Eight-to-ten-week-old female SCID (SHO-*Prkdc^scid^Hr^hr^*) mice with an average weight of 20–22 g from the Center for Genetic Resources of Laboratory Animals at the Institute of Cytology and Genetics SB RAS were used. Mice were housed in groups of 0–8 individuals in plastic cages with free access to food and water; daylight conditions were normal.

### 4.5. Silencing Activity Assay in KB-8-5 Xenograft Tumors in SCID Mice after Intravenous Administration

Tumors were initiated in mice by inoculating 106 KB-8-5 cells in 100 μL of saline solution subcutaneously into the right side of the mice and were allowed to grow to approximately 50 mm^3^ volume. Three mice per group were IV-injected with 7.5 μg/g cholesterol-conjugated siRNA, and mice were sacrificed after 4 days. The tumors were excised and cut into 100–200 mg sections; total RNA was isolated from each section with TRIzol reagent (Invitrogen, Carlsbad, CA, USA) according to the manufacturer’s protocol. Synthesis of cDNA and PCR was carried out with M-MuLV-RH and HS-qPCR reagents (Biosan, Novosibirsk, Russia) using CFX96 (Bio-Rad Laboratories Inc., Hercules, CA, USA). The amount of *MDR1* mRNA was normalized to the amount of HPRT mRNA used as an internal standard. To assess the mRNA level of the genes, the following primers and probes were used:

hMDR1 forward: 5′-CCGATACATGGTTTTCCGATCC-3′,
hMDR1 reverse: 5′-CAGCAAGCCTGGAACCTATAG-3′,
hMDR1 probe: ((5,6)-FAM)-5′-AACTTGAGCAGCATCATTGGCGAG-3′-BHQ1,
hHPRT forward: 5′-TGCTGAGGATTTGGAAAGGG-3′
hHPRT reverse: 5′-ACAGAGGGCTACAATGTGATG-3′,
hHPRT probe: ((5,6)-Rox)-5′-AGGACTGAACGTCTTGCTCGAGATG-3′-BHQ2,

The relative level of gene expression was calculated using the Bio-Rad CFX software version 3.1 (Bio-Rad Laboratories Inc., Hercules, CA, USA).

### 4.6. Statistical Analyses

The variables were expressed as the mean ± standard deviation (SD). The data were analyzed with the Student’s *t*-test or Mann–Whitney U test. The differences between the values were considered statistically significant at *p* < 0.05. The statistical package STATISTICA, version 10.0, was used for analysis.

## Figures and Tables

**Table 1:** Oligoribonucleotide sequences.

Designation ^1^	Sequence 5′-3′ ^2^
MDR1 S	GGCUUmGACmAAGUUmGUmAUmAUmGG
Ch(6)-MDR1 S	Ch(6)-GGCUUmGACmAAGUUmGUmAUmAUmGG
MDR1-(n)Ch S, *n* = 3, 8, 15	GGCUUmGACmAAGUUmGUmAUmAUmGG-(n)Ch
MDR1_−2G-(n)Ch S, *n* = 3, 8, 15	GGCUUmGACmAAGUUmGUmAUmAUm-(n)Ch
MDR1_FM S	GmGmCmUmUmGmAfCmAfAfGfUmUmGmUmAmUmAmUmGmGm
MDR1 AS	AUmAUmACmAACUUmGUCmAAGCCmAA
MDR1_FM AS	AmUfAmUmAmCfAmAmCmUmUmGmUmCfAmAfGmCmCmAmAm
SCRm AS	CmAAGUCUCGUmAUmGUmAGUmGGUU
SCRm S SCR_FM_AS Ch(6)-SCR_FM S	CCmACUmACmAUmACGAGACUUmGUU CmAfAmGmUmCfUmCmGmUmAmUmGmUfAmGfUmGmGmUmUm Ch-CmCmAmCmUmAmCfAmUfAfCfGmAmGmAmCmUmUmGmUmUm

^1^ Ch(6)—cholesterol residue attached to 5′ end of sense strand via hexamethylenediamine linker; -(n)Ch cholesterol residue attached to 3′-end of sense strand via serinol-based linker (in -(3)Ch); serinol-based linker extended with aminohexanoic acid (in -(8)Ch) or hexaethylene glycol-based linker, extended with serinol (in -(15)Ch). ^2^ Cm, Am, Gm, and Um—2′O-methyl analogs of C, A, G, and U, respectively; Cf, Af, Gf, and Uf—2′O-fluoro analogs of C, A, G, and U respectfully; Ch—Cholesterol.

**Table 2:** The value of the concentration at which the MDR1-GFP gene expression decreases by 50% (IC_50_) in KB-8-5-MDR1-GFP cells after transfection by Lipofectamine.

Designation	IC_50_, nM ^1^
siMDR1	4.4 ± 1.9 ^#^
siMDR1_AS_FM	1.2 ± 0.3 ^##,^**
siMDR1_FM	0.5 ± 0.3 ^##,^**
Ch(6)-siMDR1	15.3 ± 5.7 *
Ch(6)-siMDR1_AS_FM	3.3 ± 2.2 ^#^
Ch(6)-siMDR1_FM	0.3 ± 0.1 ^##,^**

^1^ Difference obtained using Student’s *t*-test from siMDR1: *—*p* < 0.05, **—*p* < 0.01, difference from Ch(6)-siMDR1: ^#^—*p* < 0.05, ^##^—*p* < 0.01.

## Data Availability

Data are contained within the article.
